# Assessing individual head and neck squamous cell carcinoma patient response to therapy through integration of functional and genomic data

**DOI:** 10.1038/s41598-025-03111-7

**Published:** 2025-06-05

**Authors:** Daniel Bottomly, Chase Mathieson, Myles Vigoda, Sophia Jeng, Nathaniel Evans, Ashley Anderson, Aurora Blucher, Aletha Lesch, Christina Zheng, Ted Laderas, James Jacobs, Molly Kulesz-Martin, Shannon McWeeney

**Affiliations:** 1 OHSU Knight Cancer Institute, Portland, OR 97239 USA; 2 OHSU School of Dentistry, Portland, OR 97239 USA; 3 Michigan State University College of Osteopathic Medicine, East Lansing, USA; 4 Program in Biomedical Sciences, Anesthesiology and Perioperative Medicine, School of Medicine, Oregon Health & Science University, Portland, OR 97239 USA; 5 Recursion Pharmaceuticals, Salt Lake City, UT USA; 6 Department of Dermatology, Oregon Health & Science University, Portland, OR 97239 USA; 7 Fred Hutch Cancer Center, Seattle, WA USA; 8 Randall Children’s Cancer and Blood Disorders Program at Legacy Emmanuel, Portland, OR USA; 9 Department of Cell, Developmental and Cancer Biology, Oregon Health & Science University, Portland, OR 97239 USA; 10 Division of Bioinformatics and Computational Biology, Department of Medical Informatics and Clinical Epidemiology, Oregon Health & Science University, Portland, OR 97239 USA; 11 Division of Oncological Sciences, Oregon Health & Science University, Portland, OR 97239 USA

**Keywords:** HNSCC, Antitumor drug screening assays, Multi-Omics, Computational biology and bioinformatics, Cancer genomics, Head and neck cancer

## Abstract

Even though head and neck squamous cell carcinoma (HNSCC) is the seventh most common cancer worldwide, there are only two PD-1 targeted immunotherapies (pembrolizumab and nivolumab) and one tumor intrinsic EGFR targeted therapy (cetuximab) that are FDA approved for treatment of HNSCC. Taking advantage of a high throughput inhibitor assay and computational tools originally showing success in leukemia, we designed and employed HNSCC-specific inhibitor panels that capture the diversity of aberrational pathways in HNSCC to test viable cells derived from patients’ HNSCC tumors. This provides a functional context to the multi-omic readouts conducted on these samples (mutations, protein expression and copy number alterations). In addition to generating these deeply characterized functional genomics datasets, we also developed additional visual analytics that have the potential to provide greater insight into HNSCC drug response patterns and potentially aid precision oncology tumor boards in evaluation and assessment of effective targeted therapeutic agents.

**Supplementary Information:**

The online version contains supplementary material available at 10.1038/s41598-025-03111-7.

## Introduction

Head and neck squamous cell carcinoma (HNSCC) is the seventh most common cancer worldwide^1^, with approximately 58,450 new cases and 12,230 deaths estimated in the United States for 2024^2^. The gradual increase in survival has been outpaced by the rising incidence of HNSCC^3,4^. Tumor heterogeneity continues to impede molecular characterization and subsequent improvement in outcomes^5,6^. Surgical treatment often results in comorbidities such as nerve pain, impairment in eating and vocalization and overall diminished quality of life^7,8^; whereas the efficacy of nonsurgical options such as radiotherapy and cytotoxic chemotherapy is limited by inherent and acquired resistance^5,6^. Precision therapies for HNSCC could spare tissue destruction, improve quality of life, and extend survival. However, in the United States this is currently limited to monoclonal antibodies against EGFR (cetuximab) and PD-1 (pembrolizumab and nivolumab)^9^. While an improvement over classic chemotherapy, the efficacy of EGFR targeting is not reliably predictable^10^ and patients eventually develop resistance^11,12^. Furthermore, immunotherapies targeting PD-1 are only effective for a minority of patients^13–15^ and biomarkers for predicting response are lacking^16^.

While large databases exist for HNSCC patient samples^17^ – including gene expression and mutation data – the majority of mutations and differentially expressed genes are of unknown functional significance. The genomic complexity of HNSCC has made it difficult to tie 'omics data to effective treatments. To address this, we established a deeply characterized HNSCC cohort with the goal of elucidating the drivers of drug response in HNSCC. Characterization of these patient samples included multiple genomics assays including whole exome sequencing, RNA sequencing, copy number arrays and reverse-phase protein arrays as wells as evaluation with a panel of small-molecule inhibitors and natural products. Our previous work focused on the development of an HNSCC-specific inhibitor assay through the identification of HNSCC-specific biological pathways that could potentially be targeted by current FDA approved cancer drugs^18^. The integration of high-throughput tumor drug response data with genomic characterization has proven useful in leukemia^19^, but similar study of HNSCC has been restricted to cell lines as part of multi-cancer^20,21^ and HNSCC specific studies^22–24^. This study highlights the feasibility of this approach in patient-specific cells tumor cell models in HNSCC. Here we provide a clinically-relevant dataset and highlight patient-specific analyses that potentially could be used for tumor-boards based on the integration of genomic and drug response data that can be used to help guide development of larger scale HNSCC studies.

## Results

### Clinical and genomics characteristics

The cohort consists of twenty patients enrolled at the time of surgical treatment for primary (*n* = 14) or first recurrence (*n* = 6) of HNSCC (Supplementary Table S1). The anatomical sites represented include the oral cavity (*n* = 12), larynx (*n* = 4), and oropharynx (*n* = 3) as well as a single rare (biologically and epidemiologically distinct) tumor of the maxillary sinus. No patient is known to have had more than one diagnosis of recurrent (*n* = 1) or metastatic (*n* = 4) HNSCC following the diagnosis of the cancer collected. Multiple patients (*n* = 5) had histories of other cancers prior to their first diagnosis of HNSCC, but only one (10139) was diagnosed with a non-HNSCC cancer (cutaneous SCC-MOHS) following their first diagnosis of HNSCC.

In total, we obtained results for at least one genomics-based assay for all 20 patients as summarized in Supplementary Table S2.

### Comparative analyses with TCGA-HNSC

We obtained tumor tissue from 16 of the patients for whole exome sequencing, with whole blood to serve as a control for somatic genotyping. In The Cancer Genome Atlas HNSCC cohort (TCGA-HNSC)^17^, they reported 11 significantly mutated genes. Of these 11 genes we found mutations in 8 genes in our HNSCC cohort. When we compared frequencies between TCGA-HNSC and our cohort we found that 5 genes had < 10% difference. The most similar frequencies were for TP53 and NOTCH1, NSD1 and PIK3CA which differed by 1%, 1%, 3% and 6% respectively (Supplementary Fig. S1a). While TP53 and PIK3CA are also significantly mutated in other TCGA cohorts from Pan-Cancer analyses, NOTCH1 and NSD1 appear to be significantly mutated only in TCGA-HNSC^25^. We further assessed array-based copy number alterations (CNAs) on 8 / 16 patient samples, observing overall higher frequencies of the putative driver recurrent CNA driver genes than found in TCGA-HNSC (Supplementary Fig. S1b). The closest match was CDK2NA which was observed in 25% of our HNSCC cohort and 28% of TCGA.

### Expression subtyping of the cohort

**Fig. 1:**
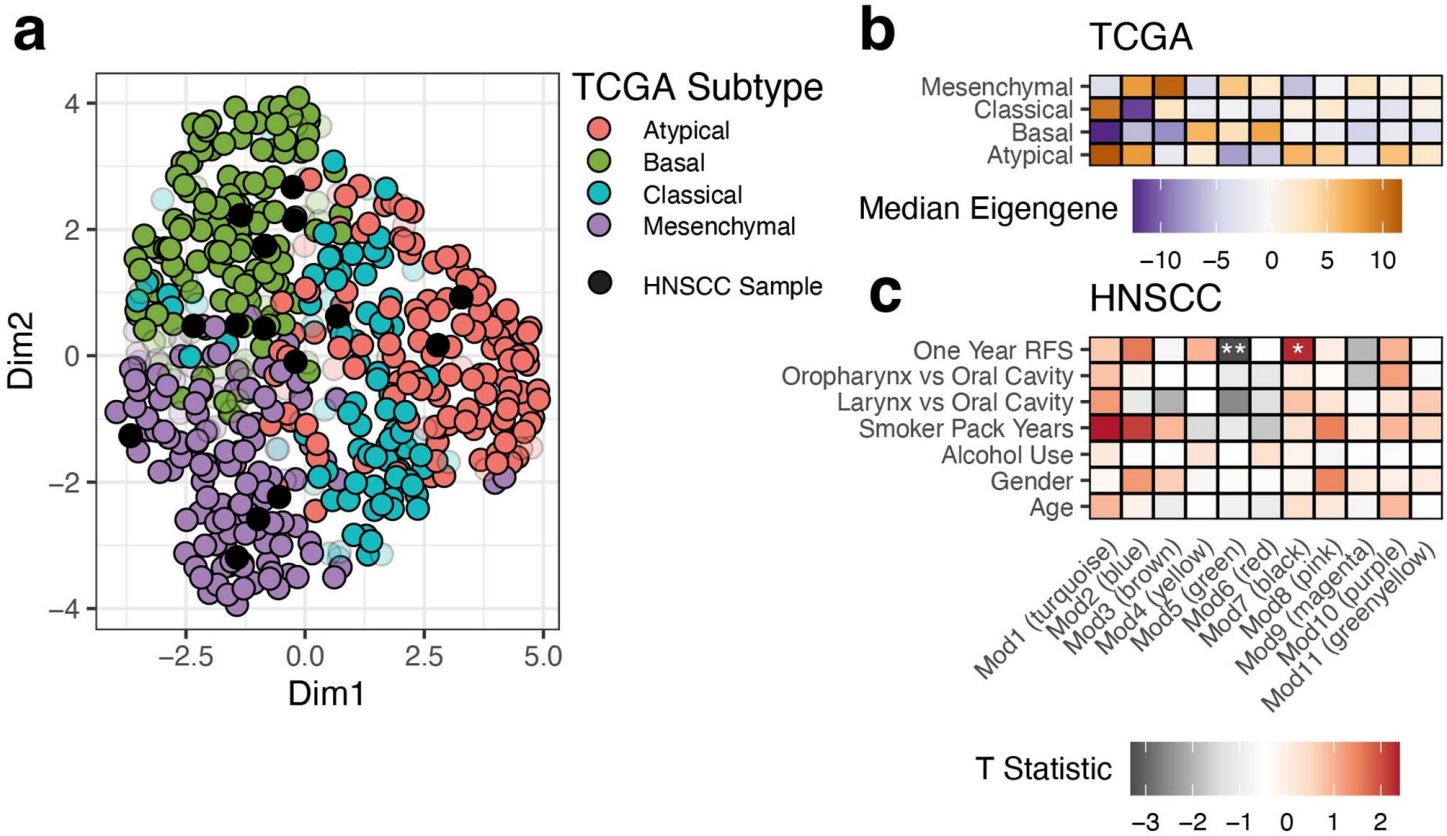
**Weighted Gene Co-expression Analysis provides biological context to known HNSCC subtypes and correlates with clinical covariates in the HNSCC cohort.** RNASeq from the HNSCC cohort tumor tissue samples were combined with TCGA and the four main subtypes were called for both cohorts. (**a**) A UMAP of expression data from the 643 genes used for determining subtypes is shown for TCGA with black dots indicating the HNSCC cohort samples. Samples that could not be confidently called (see “Methods”) are shown as transparent circles. (**b**) A heatmap of the median PC1 score (termed eigengene) in TCGA–HNSC is shown for each module (X-axis) and subtype (Y-axis) indicating the overall expression trend for genes in the module. (**c**) T-Statistics from specified comparisons (Y-axis) are shown for each module (X-axis). Stars indicate significance of the corresponding T-test where the unadjusted P-value < 0.001 is ***, < 0.01 **, < 0.05 * otherwise no significance. For the directionality of the tests not specified on the plot: One Year RFS–Yes vs. No, Smoker Pack Years–[0,15] vs. (59,100], Alcohol Use—Heavy vs. Minimal, Gender—Female vs. Male, Age–(40,62] vs. (62,90].

Additionally, we performed RNASeq on 15 patient samples, most of which were also characterized with DNA-based analyses (14 / 15; 93%). We obtained and ultimately included 499 samples from the TCGA-HNSC project from the Genomic Data Commons (GDC)^26^ and harmonized our samples with them (see “Methods”). We first evaluated our ability to assign these patient samples to four previously characterized expression subtypes (Atypical, Basal, Classical and Mesenchymal)^27,28^ that were called in a subset of the currently available TCGA-HNSC cohort^17^. We were able to re-call these subtypes with 96% accuracy in TCGA, allowing us to further annotate all available RNASeq samples in TCGA-HNSC and our HNSCC cohort. We determined that our HNSCC cohort represented all four subtypes (Fig. 1a) with both of the HPV+ patients being part of the Atypical subtype consistent with previously observations^27^.

### De-novo network analyses

To further characterize our expression data with regard to network-based signatures, we first separated the most variable 2,500 genes from the TCGA-HNSC patient samples into 12 co-expression modules using WGCNA^29^. These modules are denoted by a number and color (e.g. ‘Mod1 (turquoise)’). Note that the ‘Mod0 (grey) module’, which customarily contains genes with low co-expression, is not considered in any analyses. These modules were overrepresented for a variety of hallmark gene sets^30^ (Supplementary Fig. S2a). For each module, patients were scored by their first two principal components (PCs) and after harmonization, our HNSCC cohort clustered with the TCGA samples (Supplementary Fig. S2b). The first PC is termed the module eigengene^31^ and can represent the trend of the expression of the genes within the module. Using the median of the module eigengene values in TCGA, we found that at least one module was highly expressed in each HNSCC subtype (Fig. 1b). For instance, patient samples with the Mesenchymal subtype tended to have increased expression of genes in the Mod3 (brown) module which was significantly (FDR < 0.05) associated with the epithelial-mesenchymal transition hallmark. When we compared the module eigengene values between clinical groups we found that two modules, Mod5 (green) and Mod7 (black), were marginally significant (T-test; unadjusted P-value < 0.01) and suggestive (T-test; unadjusted P-value < 0.05) respectively with one-year relapse free survival (RFS) (Fig. 1c). The modules were seen to be associated in opposite directions, with Mod5 (green) decreased in patient samples who achieve one-year RFS while Mod7 (black) was increased. The correlation with the module eigengene (kME)^31^ is a measure of membership to a given module that can be used to prioritize biomarkers and can provide additional biological relevance. A clear instance of this is the INHBA gene which is the top ranked gene by kME in the Mod5 (green) module. INHBA is involved in EMT processes^32^, with high expression previously characterized to be associated with poor prognosis in HNSCC^33^.

### Formation of tumor cell models

We established tumor cell cultures from patient solid tumor samples to facilitate inhibitor screening. Of approximately 90 cases attempted, 20 yielded epithelial tumor cultures sufficient for the inhibitor assays. The appearance of hematoxylin and eosin (H&E)-stained sections and pavement-like morphology of the corresponding patient-derived tumor cells in culture (Supplementary Fig. S3; top and bottom) were consistent with epithelial origin. Samples 10336, 10021 and 10356 had varying degrees of mesenchymal morphological characteristics, i.e. subsets of cells tending toward fusiform fibroblast cell morphology and swirls/whorls of colony morphology. To further characterize epithelial composition and ensure minimal fibroblast composition, cell lines were stained with epithelial (keratin 5 and K1/18) and mesenchymal vimentin markers as described in “Methods”. The majority of cell lines expressed keratinocyte markers and were found to contain minimal fibroblast cells (Supplementary Table S3). We further characterized keratin expressing epithelial cells as having an increased mesenchymal character if those cells were found to express the mesenchymal/fibroblast marker vimentin more strongly than the epithelial keratins (Examples of representative fibroblasts and epithelial cell lines expressing varying degrees of mesenchymal character are presented in Supplementary Fig. S4). Epithelial cells going through epithelial-mesenchymal transition (EMT) can progress to undifferentiated cells indistinguishable from fibroblasts; however, none in our cohort appeared to lose epithelial characteristics, consistent with the squamous cell carcinoma pathological assessment of the original tumor. Finally, to facilitate evaluation of drug-associated biomarkers we also generated RNASeq and reverse-phase protein array (RPPA) data for these tumor cell culture samples (Supplementary Table S2).

### Description of inhibitor assays

**Fig. 2:**
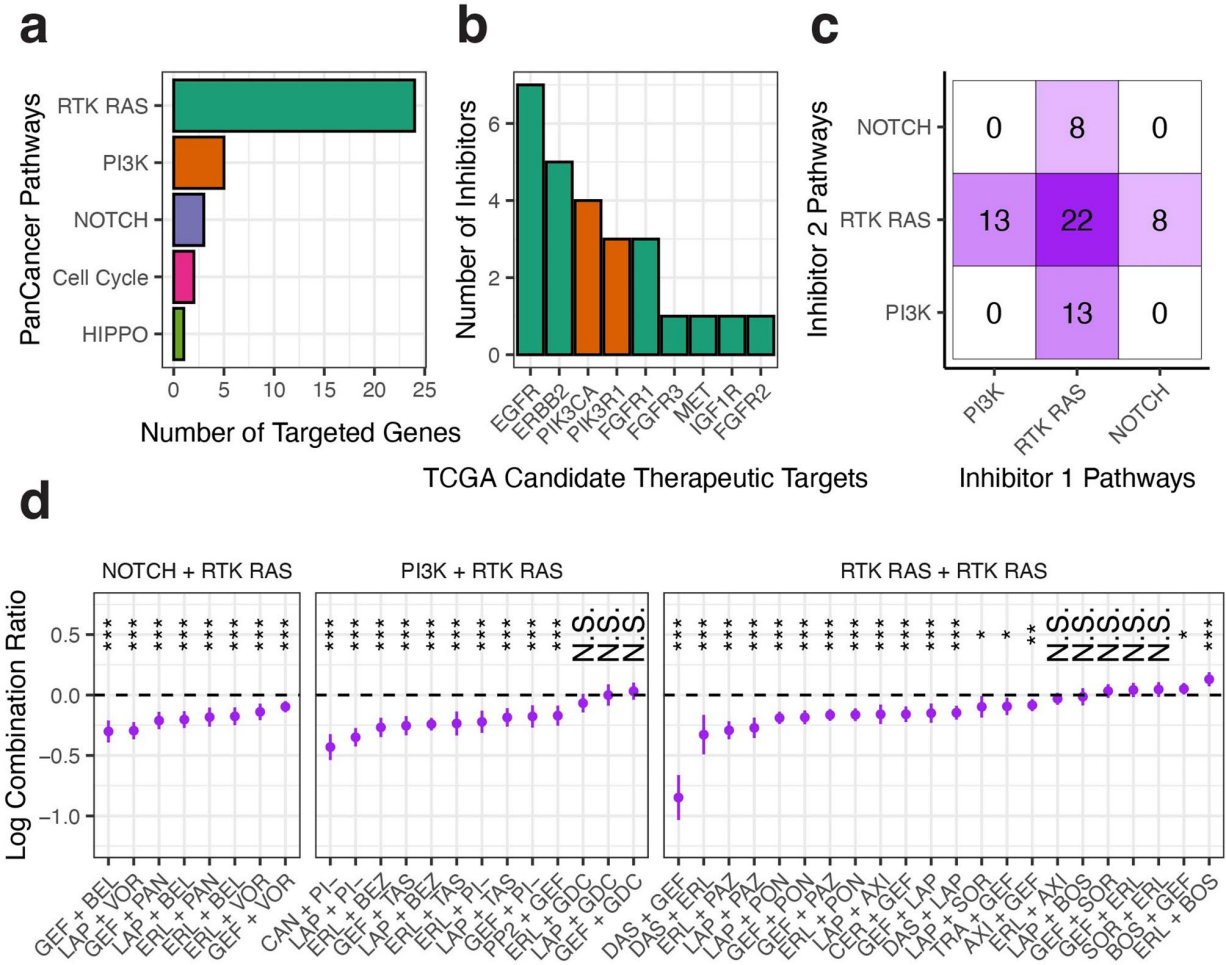
**Summary of inhibitor pathway targeting. **(**a**). A barplot indicating the number of targeted genes for each single-agent inhibitor used with the HNSCC cohort (X-axis) for each PanCancer pathway (Y-axis). (**b**) A barplot indicating the number of single-agent inhibitors used with the HNSCC cohort (Y-axis) for each candidate therapeutic targets for TCGA-HNSC. Colors correspond to the PanCancer pathways from (**a**). (**c**) Shown is a heatmap indicating the annotated co-targeting of the inhibitor combinations used in the HNSCC cohort. Text indicates the number of inhibitors with matching color intensity. (**d**). Paired t-test summary results for each drug combination (X-axis) grouped by single-agent PanCancer pathway (top). For each combination, the estimated difference (termed the log combination ratio; Y-axis) between the combination log AUC and the lowest single-agent log AUC is shown as a dot centered on a line representing the 95% confidence interval. Negative values indicate the combination is more sensitive than the corresponding single-agents. Stars indicate significance with P-value < 0.001 ***, < 0.01 **, < 0.05 * and N.S. indicating non-significance.

Tumor-derived cell lines at low passage (P1–P2) were exposed to a panel of 89 single-agent targeted inhibitors and natural products. Using our high confidence set of Targetome interactions (see “Methods”) we found that most of the covered drugs (drugs that have known drug target interactions) were concentrated within the PanCancer RTK-RAS pathway^34^ (Fig. 2a, See Supplemental Table S4 for full target annotation). This included 9 of the 18 genes considered to be candidate therapeutic targets in TCGA-HNSC^17^ (Fig. 2b).

Tumor-derived cell lines were also treated with 49 inhibitor combinations with targets similarly concentrated on combinations of RTK-RAS pathway genes (Fig. 2c). Sensitivity to each agent and combination was determined by calculating the area under the dose-response curve (AUC). Comparing the combination log AUC to the lowest single-agent log AUC for each inhibitor over all samples resulted in 31 combinations being significantly (Paired T-Test; unadjusted P-value < 0.001) more sensitive than either single-agent suggesting non-redundancy of the underlying pathways (Fig. 2d).

### Consistency of inhibitor responses with external data

**Fig. 3:**
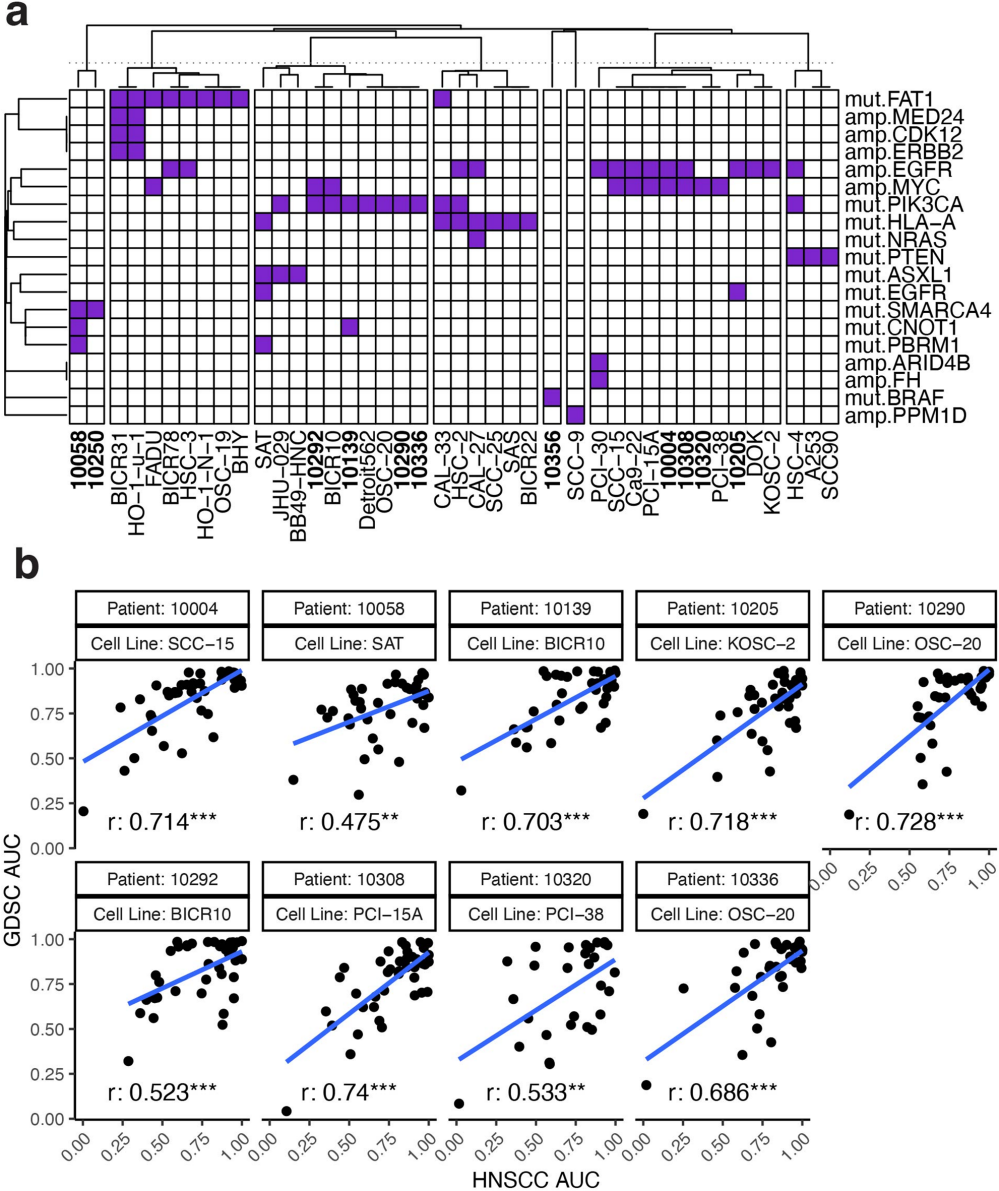
**Drug response in the HNSCC cohort is consistent with large-scale cell line screens.** Patient samples and GDSC cells lines were clustered according to alterations associated with drug response to one of the common inhibitors between the HNSCC cohort and GDSC. (**a**) Shown is a matrix plot where columns (samples or cell lines) and alterations (rows) are clustered based on their Jaccard distance. Purple indicates that the patient sample or cell line has the corresponding alteration. Only those patient samples (bold) or cell lines with at least one alteration are shown. Note that patient sample 10250 and 10356 have no matching cell lines based on their mutation profile. Pearson’s correlation was computed between the normalized AUC values for each patient sample and the AUCs of the matching inhibitors in GDSC. (**b**) Scatterplots of the AUC values for the matching drugs between the HNSCC cohort and GDSC are provided. Each scatterplot is faceted by patient sample id and the corresponding best GDSC cell line name (boxes on the top). The text indicates the Pearson’s correlation. Unadjusted significance is indicated by P-value: < 0.001 ***, < 0.01 ** < 0.05 *.

Large scale efforts such as the Cancer Cell Line Encyclopedia^20^ and the Genomics of Drug Sensitivity in Cancer (GDSC)^35^ project have evaluated drug response in many commonly used cell line models across cancer types including HNSCC. In order to evaluate consistency of our drug response data to these cell line models, we retrieved mutation, CNA and drug response data (AUC) for 42 cell lines from the GDSC project with data for at least one common drug. Although several approaches such as CELLector^36^ can find relevant cell lines to TCGA or another large cohort based on their alteration status, we wanted to match our patient samples with cell lines in a manner consistent with drug response. We first found the significant pharmacogenomic interaction gene alterations in the PanCancer and HNSC-specific analysis from Iorio et al. 2016^37^. These genes could be more relevant to drug response than those most frequently mutated. Using these pharmacogenomic interaction genes, we then evaluated the Jaccard distance from each patient sample’s mutation and or CNA profile to each cell line similar to the approach of Sinha et al. 2021^38^ (Fig. 3a). A total of 9 patient samples could be matched to one or more cell lines using this approach. Of those, 7 had significantly correlated AUC values (Pearson’s correlation; unadjusted P-value < 0.001), the remaining 2 also had strong correlations (unadjusted P-value < 0.01) (Fig. 3b). We did observe a negative trend between correlation and alteration distance (i.e. higher correlation was seen on average for lower distances; Supplementary Fig. S5) suggesting the utility of this approach.

### Exploration of inhibitor response in patients

**Fig. 4:**
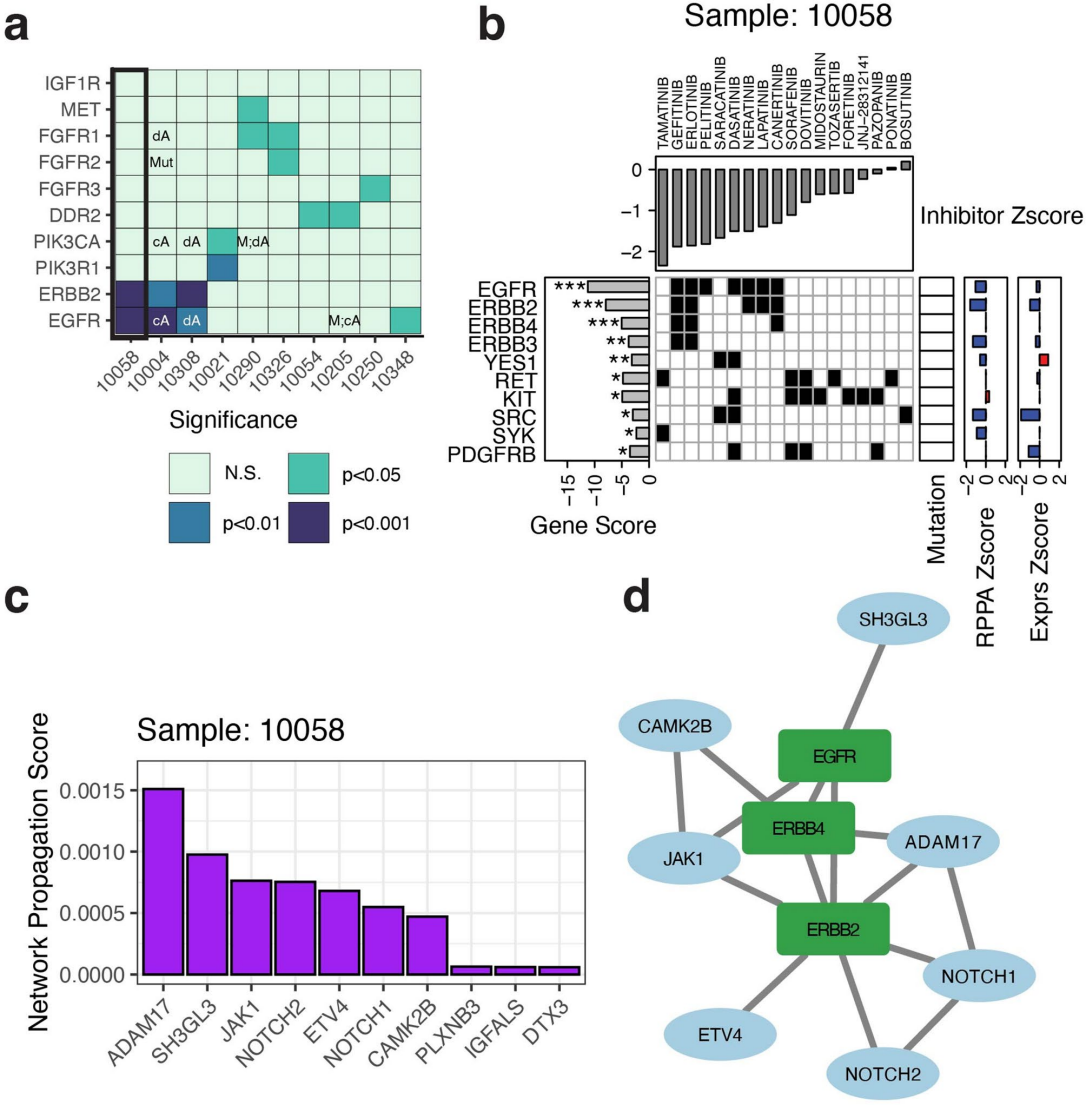
**Prioritization of genes based on drug sensitivity.** (**a**). Shown is a summary of the gene score significance for the HNSCC cohort patients relative to the TCGA candidate therapeutic target genes. The EGFR gene scored highly in three patients, two of whom had amplifications of the gene via copy number. The other patient, 10058, didn’t have copy number data. Patient 10058 had EGFR as the most significant gene target and was unlikely to have had an underlying amplification as it showed mild down-regulation of EGFR from both RPPA and expression. (**b**). Shown is the Response Card for patient 10058. Inhibitor data is shown on the top in the form of a Zscore with negative values indicating increased sensitivity. From left to right, the scores of the top genes by significance are shown along with SYK which was a target of the most significant drug. In addition, a heatmap displays the drug target data from Targetome. Finally, any mutations (black rectangle) in these genes are shown along with RPPA and expression from the tumor cell culture models after being centered/scaled to Zscore values. If available, copy number data is also displayed. (**c**) Using a network propagation approach we prioritized the patient’s somatic mutations and the results are displayed as a barplot where the resulting prioritization score is on the Y-axis and corresponding gene is on the X-axis. (**d**) The mutations that scored highly in the prioritization (blue ovals) were seen to interact with EGFR and the other significant gene targets (green rectangles) either directly or indirectly in a network context.

We first devised a gene scoring approach for our AUC-based drug response as previous methods have used IC50 values^39^. Our statistic was based on the sum of centered/scaled drug AUC values (i.e. Zscores) for each gene and significance was determined using a permutation test per gene. Negative values of these gene scores indicate the degree of sensitivity to a given drug based on the inhibitor distributions. If a mutation or copy number alteration occurred in a gene with a highly negative score it suggested etiological relevance for that patient with respect to their drug response profile. We further refined our set of copy number alterations by comparing the concordance/discordance of the values with the cell culture-based RNASeq and RPPA directionality. For instance, if RNASeq and/or RPPA was available and both had a positive Zscore, then an amplification was considered a concordant amplification (cA). Likewise, a discordant amplification (dA) would have a negative Zscore for the available cell-based data. When looking at the TCGA candidate therapeutic targets we found that only in the case of EGFR amplifications did a mutation or CNA co-occur with a significant gene score (Permutation Test; unadjusted P-value < 0.001; Fig. 4a). Patient 10058 was of particular interest due to EGFR being both significant (Permutation Test; unadjusted P-value < 0.001) but not having a corresponding alteration. To further explore the direct relationships between drug response, alterations and genes, we created an approach to visualize these data called ‘Response Cards’. The Response Card for patient 10058 showed no corresponding direct alteration for any of the significant or suggestive genes (Permutation Test; unadjusted P-value < 0.001 or 0.05 respectively; Fig. 4b), indicating a potential indirect association with an altered gene. Even though this patient did not have CNA data, we would still expect a putative CNA to have a high or low (> abs(2)) RNASeq or RPPA Zscore; this was not observed to be the case. To prioritize the remaining mutations based on gene interaction network proximity, we used a random walk with restarts based approach^40^ in conjunction with the Reactome FIViz gene interaction network^41^. We found 7 mutations that occurred in genes considered to be neighbors of EGFR, ERBB2 or ERBB4, the three most significant genes by score (Fig. 4c, d). This combination of studying both direct and indirect alterations allows for a more effective interpretation of the integrated genomics and functional data via the response card and adds considerable value to this dataset.

## Discussion

HNSCC represents a genomically heterogeneous malignancy in which mutation and copy number alteration data are not adequate for guiding the development of personalized targeted therapy. Indeed, histopathologically indistinguishable HNSCC samples are likely to have distinct somatic mutational abnormalities^42^ leading to clinical uncertainty regarding the best treatment options. This study highlights the feasibility and potential utility of functional screening in solid tumors like HNSCC with the assays and methods originally designed for hematological malignancies. By integrating genomic alterations with drug response data from patient-derived tumor cell cultures, our dataset provides the potential to achieve a more thorough understanding of the oncogenesis of individual HNSCCs.

When comparing our cohort to TCGA-HNSC we observed similar mutation distributions, with differences likely due to much smaller sample size in our HNSCC cohort. Additionally, the discordance in CNA frequency can also be explained by cohort size as well as differences in array platform (i.e. Affymetrix CytoScan HD vs. SNP 6.0 chips used in TCGA), as well as the resulting methodological differences. For instance, the Affymetrix CytoScan HD array has fewer probes than the SNP 6.0 and it has been seen to have poor performance in validating known CNVs ≤25 kb, suggesting that it is better suited for the detection of larger CNVs^43^. The same study also found that there was often high discordance depending on the software and parameterization used. Additionally, we found that our cohort also represented all four expression subtypes. The expression data could be further explored by using co-expression-based methods, which divided the most variable genes into modules. Two of these modules were seen to be associated with one year recurrence-free survival providing the opportunity to evaluate them as prognostic biomarkers in the future with larger datasets. Our inhibitor data were consistent with previously generated cell line data from GDSC. This further indicated the value of our dataset and approach since the data were derived from actual patient samples and are therefore more clinically relevant. When prioritizing genes with respect to drug response, we note that the genomic alterations were rarely in the targeted genes and therefore network-based approaches are necessary to provide a more complete picture of the genes that could underlie observed differences in drug response. Complicating these analyses is the potential for the heterogeneity of the cell lines to become masked by the competitive nature of cell growth in culture. Over time, evolution of the cell population can alter the functional (drug response) and genetic heterogeneity of the cultures relative to the original tumor. This is due to the fact that the fastest growing epithelial cell populations are arbitrarily selected as passage numbers increase. External heterogeneity can also develop from new mutations that can accumulate in culture over time^44,45^. We sought to mitigate this effect by collection of aliquots for genomic and functional studies from primary cultures directly or early passages (1–2). Future application of integrated analyses could also utilize drug response data from implantable microdevices in human tumors prior to surgery^46^, replacing tumor cell culture all together.

This dataset provides the first functional context for HNSCC in a representative cohort with in-depth characterization and epithelial tumor cell morphology in culture. More importantly, it provides a framework for the experimental and computational approaches to scale this work in larger cohorts and future studies. The patient-specific “Response Card” and corresponding network-based prioritization not only helps maximize the available information from this cohort but also provides a foundation for precision oncology trials in HNSCC. These tools in the context of SMMART trials being carried out at our institute^47^ will allow for the integration of intrinsic and extrinsic tumor targeting as we move closer to the goal of personalized targeted therapy for HNSCC.

## Methods

### Ethics

This study had the approval of the Institutional Review Board at Oregon Health and Science University. All patients gave informed consent to participate in this study and all samples were de-identified. All experiments and methods were performed in accordance with relevant guidelines and regulations.

### Patient sample collection and clinical annotation

Tumor, uninvolved oral mucosa, and blood samples were collected from HNSCC patients undergoing surgical resection. Tissues were collected from patients undergoing surgery as the initial treatment for either a primary or first recurrence of HNSCC. Relapse-free survival was measured from the end of the primary (surgical) treatment to the date of next diagnosis by biopsy. Following resection, uninvolved and tumor tissues were each immediately divided into four pieces treated as follows: 20 mg of each tissue were placed in RNAlater (Invitrogen) for 24 h at 4 C prior to storage at -80 C. 20 mg were flash frozen in liquid nitrogen and stored at -140 C. The remaining tissues were divided between culture and 24-hour formalin fixation for paraffin embedding. Whole blood was frozen at -80 C in EDTA treated blood collection vacutainers.

### Cell culture and immunostaining

Patient-derived cell cultures were utilized at low passage P1 – P2 (with first replating of primary cultures defined as passage number 1) so as to more closely reflect the epithelial tumor heterogeneity present in the patient’s tumor^44,45^. Tumor tissue was transported to the lab in transport media consisting of DMEM/F12 Media (Gibco, 11320082) supplemented with 2x antibiotic-antimycotic (Gibco 15240112) and 5% iron-supplemented bovine calf serum (Cytiva, SH3007203). Tissues were minced to approximately 1 mm pieces and grown in tissue-culture treated dishes (Corning, 430293) and roller bottles (Corning, CLS 430195). Roller steady state culture techniques were used to more closely replicate in-vivo conditions. Tissues were cultured in DMEM/F12 Media supplemented with 5% BCS, 1x penicillin-streptomycin (Gibco, 15070063), 5 µg/mL insulin (Sigma, 19278), 10 µg/mL epidermal growth factor (Gibco, PHG0311), 0.18 mM adenine (Sigma, A2786), 0.1 nM cholera toxin (Sigma, C8052), 0.02nM Triiodo-L-thyronine (Sigma, T6397), and 0.4 µg/mL hydrocortisone (Sigma, H0888). Fibroblasts were periodically removed from epithelial culture via cell scrapers and differential trypsinization (Trypsin-EDTA, Gibco, 25200114). All cells were passaged between 60 and 80% confluence. Cell culture methods for adherent epithelium were used^48^. For examination of epithelial mesenchymal transition (EMT), cells were formalin fixed and subjected to indirect immunofluorescence using primary antibodies to epithelial-specific intermediate filament markers Keratin 5 (K5; abcam-ab52635, rabbit), Keratins 8 and 18 (K8/18; Fitzgerald 20R-Cp004, guinea pig), and mesenchymal intermediate filament marker vimentin (abcam ab8978, mouse). Nuclei were stained with DAPI (405 nm). Secondary antibodies for K8/18 were Goat anti-Guinea Pig IgG with Alexa Fluor™ 488 (A-11073), for vimentin were Goat anti-Mouse IgG with Alexa Fluor™ Plus 647 (A32728), and for Keratin 5 were Goat anti-Rabbit IgG with Alexa Fluor™ 594 (A-11037). No primary antibody replicate cultures served as primary antibody controls.

Ten sets of four superimposable sub-images (CY5, DAPI, GFP, and TxRed channels) were captured for each cell line; five sets of the cells treated with primary antibodies and five of the no-primary controls. Images were captured with an EVOS FL Color Imaging System (AMEFC4300). ImageJ was used to quantify the relative intensity of each channel associated with each cell within each image. Fibroblastic cells were distinguished from epithelial cells based on their sole expression of vimentin. Epithelial cells (cells expressing keratin) with a vimentin signal more intense than the combined signal of K8/18 and K5 were described as having increased mesenchymal character.

### Inhibitor assays

Drugs were selected for our inhibitor panel based upon initial responses of HNSCC and cutaneous SCC to a panel designed for leukemia^19,49^. The functional screening platform, which requires only the tumor cells, was developed over 10 years ago and has been validated for FDA clinical trials (under an IDE or as a correlative readout). Tumor cell lines were exposed to seven 3-fold serial dilutions of 89 single agents and 49 agent combinations within their first two passages. Adherent epithelial cell lines were trypsinized and strained at 70 μm (Corning, 431751) to achieve a near single cell suspension. Cells suspended in DMEM/F12 Media, 5% BCS, and 0.5x antibiotic were plated in 384 well plates (Greiner, 781182) containing pre-aliquoted inhibitors dissolved in 46nL DMSO per well, to a final volume of 48uL per well. Cells were plated at 4000 cells per well using a Multidrop Combi Reagent Dispenser. Following a 72-hour incubation at 37 deg C, 5uL of MTS (a colorimetric assay of metabolic activity used to infer cell viability) (Promega, G3581) was added to each well and allowed to incubate for one hour. For metabolically slow cell lines, incubation time was extended to ensure absorbances fell within the established linear range. Following incubation, absorption at 490nM was measured with a Biotek Synergy H1 Microplate Reader. Values on each plate were normalized to the average of 46 negative control wells containing only DMSO, media, and cells. Wells containing a lethal mixture of flavopiridol, staurosporine, and velcade provided blank (no cell) data.

#### Formation of response AUC values

Data were pre-processed following the workflow from Tyner et al. 2018^19^ with the following modifications. Data were first harmonized by calculating an ordinary least squares (OLS) regression for those runs with within-panel replicates after applying a ceiling of 1 and a floor of 0 for the normalized viability. If the AUC values showed an increase (i.e., greater cell viability with higher inhibitor concentration), those curves were removed. Any negative cell viability values that had a floor of 0 applied were flagged. The maximum change in AUC amongst the replicates was noted and those runs with differences > 1 were flagged. Remaining within-plate replicates had their normalized viability averaged and subject to a ceiling of 1 and a floor of 0. An additional set of OLS AUCs was computed for sample-inhibitor pairs run on multiple panels. The maximum change in AUC amongst the across-panel replicates was noted and those runs with differences > 0.75 were flagged. The within and across plate replicates were then averaged together with a ceiling of 1 and a floor of 0. A probit regression was then fit to all possible run groups. The AUC values were rescaled relative to the range of log10 concentration values when compared with the GDSC AUC values and when comparing single-agents with drug combinations. These data are publicly available at https://biodev.github.io/HNSCC/.

#### Annotation of drug targets

In order to establish a high-quality list of targets we utilized the Cancer Targetome^50^. We required all targets to have assay values (termed Tier III) and further required these values be less than 100 nM. To maximize coverage while minimizing inclusion of lesser characterized targets, drugs-target interactions were categorized based on the number of supporting publications (> 1, 2 or 3). For each drug, only gene targets that were part of the highest category were kept. Note that since our drug screen also includes natural products, we interrogated the natural product database based on Targetome^51^, only finding one interaction meeting the 100 nM threshold (Coumarin -> CA14). However, since there was a large discrepancy in assay values between the two available references (48 vs. > 200000 nM), we decided to not include it.

### Whole-exome sequencing

DNA was isolated from either previously snap-frozen, FFPE preserved or RNAlater treated tumor tissue. Patient matched controls were derived from EDTA treated whole blood or snap frozen uninvolved tissue. Preparation for sequencing was performed using either the DNeasy blood and tissue kit, QiaAmp DNA Mini Kit, AllPrep DNA/RNA/Protein Mini Kit (Qiagen) or NucleoSpin Tissue kit. Whole-exome sequencing was performed using Illumina’s Nextera Rapid Capture Enrichment kit on an Illumina HiSeq 2500 by the Oregon Health and Science University Massively Parallel Sequencing Shared Resource. Preprocessing was performed using an implementation of the GenomeAnalysisToolkits Best Practices^52^. Finally, Mutect2^53,54^ was used to identify candidate somatic mutations in the tumor samples relative to their matched normal samples^55^. We annotated the candidate somatic mutations using the Ensembl Variant Effect Predictor (v99.1)^56^. We only kept mutations that passed the Mutect2 filter, had a predicted non-synonymous effect or were indels, had a gnomAD^57^ allele frequency < 0.01 and were not tolerated in SIFT^58^, benign in PolyPhen^59^ or were considered clinically pathogenic in ClinVar^60^. Finally we removed any potential false positive mutations/genes using a previously curated list^61^.

### RNASeq

For tumor tissue and uninvolved (normal) samples, RNA was isolated from either Snap Frozen Tumor Tissue samples or from samples preserved in RNAlater using either the RNeasy mini Kit (Qiagen) or the AllPrep DNA/RNA/Protein Mini Kit (Qiagen). For tumor cell cultures, RNA isolation was performed using the RNeasy mini Kit (Qiagen).

Sequencing was performed at two locations, either at OHSU or OSU:

#### OHSU sequencing

At the Oregon Health and Science University Massively Parallel Sequencing Shared Resource, RNA quality was first assessed using the Agilent 2100 Bioanalyzer. Libraries were then prepared using the TruSeq Stranded mRNA kit (Illumina). Briefly, 100ng of total RNA was converted to cDNA using random hexamers. Synthesis of the second strand was done with the addition of dUTP, which enforced the stranded orientation of the libraries by blocking amplification off the second strand during the first round of PCR. Amplified libraries were profiled on the 4200 TapeStation (Agilent). Libraries were quantified for sequencing using an NGS Library Quantification Kit (Roche/Kapa Biosystems) on a StepOnePlus Real Time PCR Workstation (Thermo/ABI). Sequencing was performed on an Illumina HiSeq 2500 Instrument.

#### OSU sequencing

Libraries were prepped using Wafergen PrepX PolyA mRNA Isolation Kit followed by Wafergen PrepX RNA-Seq for Illumina. The preps were done on the Wafergen Apollo 324 liquid handler. Final RNAseq libraries were quantified by Qubit fluorometer using the Qubit dsDNA HS Assay. Then the libraries were run on the Agilent BioAnalyzer 2100 using the high sensitivity DNA chip to check sizing. Finally, all libraries were quantified by qPCR using the ABI 7500 fast instrument using the KAPA Biosystems Library Quantification Kit. Samples were normalized and pooled, then run on 6 lanes of the Illumina HiSeq 2000, 100 bp paired end.

#### Preprocessing

Alignment and counting of the reads was performed using the protocol published by the GDC^26^. Equivalent gene count data for TCGA-HNSC were also downloaded from the GDC for 520 patient samples derived from the primary tumor. Based on an initial expression filtering using ` filterByExpr` function in edgeR^62^ and transformation into counts per million, we removed 21 samples whose median expression was less than 2 standard deviations below the mean. Finally, for each collection of samples (tumor, normal, cell culture and TCGA-HNSC) we first filtered counts, normalized using the Trimmed mean of M-values methodology^63^ and finally computed the log2 RPKM using the functionality in edgeR.

#### WGCNA

Using the filtered and normalized log2 RPKM data, we first limited genes to the common set between the TCGA-HNSC and HSNCC cohort samples. We used ComBat^64^ to adjust for the batch effects between the different sequencing runs in the HNSCC cohort and the TCGA-HNSC cohort using TCGA-HNSC as the reference. The effectiveness of this approach was verified using BatchQC^65^. Limited to only the 499 TCGA samples, we evaluated a range of parameters for WGCNA including the number of variable genes, power and four variations of key module detection parameters using a combination of subsampling as well as assessment of module quality^66^. Based on these results, we chose to use the 2,500 most variable genes and create a signed-hybrid network using ‘bicor’ correlation^67^ with maxPOutliers = 0.1, power = 3, deepSplit = 2, detectCutHeight = 0.995, minModuleSize = 30, pamStage = T. For the HNSCC cohort, the principal component values used for the eigengenes were generated using ‘prcomp’ in R after centering and scaling based off the per-gene mean and standard deviation of the TCGA data. Enrichment of the module genes was computed using clusterProfiler^68^, for each module, limiting the universe to the 2,500 most variable genes.

#### Subtype calling

We used a variation of a prior method implemented in breast cancer^69^. We first downloaded the centroids used in the TCGA-HNSC publication as well as their published prior calls^70^. Next, we median centered the expression data by gene and computed the Pearson’s correlation between each centroid and the expression of the common genes. The subtype with the best correlation was assigned. This was the value used for assessing the performance in TCGA-HNSC. However, as seen in Fig. 1a if the correlation was negative or the second-best correlation was too close (< 0.1) we flagged these calls as indeterminate.

### Copy number alterations

Copy number was assessed for eight cases with sufficient DNA after WES using the same samples. Copy number analysis was performed by the Oregon Health and Science University Gene Profiling Shared Resource, using a CytoScan HD Array Kit on a GeneChip™ Scanner 3000 7G System. First RawCopy^71^ was used to generate log ratios and B-allele frequencies for the tumor and matched normal samples relative to GRCh37. ASCAT^72^ was then used produce segmented allele-specific copy number for the tumor samples. The segments were then overlapped with Gencode v19^73^ protein coding genes. The total copy number was determined by taking the sum of the major and minor alleles. We median centered the total copy number for each sample and assigned each gene the largest (based on absolute value) centered copy number value. Amplifications and deletions were called based on genes having a centered value > 1 and < − 1 respectively.

### Reverse phase protein array

Cell cultures were grown to 70–90% confluence in 6-well plates. Following protein extraction with lysis buffer (purchased; MD Anderson), lysate was further diluted in lysis buffer and 4x SDS buffer (purchased; MD Anderson) to 1.5ug/ul. Samples were stored at -20 C before delivery to MD Anderson. Core protocols for RPPA data generation, processing and normalization were utilized for the data^74^. For all analyses, we used the 278 antibodies considered to be validated^75^. Analyses were performed using log2 transformed normalized linear data.

### Integrative analyses of inhibitor data

#### Drug target score

The database of drug-target interactions was structured as a *g* x *d* matrix $\:T=\left[{t}_{ij}\right]$ where $\:{t}_{ij}$ = 1 if gene *i* was a target for drug *j* and 0 otherwise. Next, we defined a *d* x *n* matrix *X* consisting of up to *d* AUC values for each of the *n* patients. For each drug *j* we first centered and scaled each sample *k* as $\:{(X}_{jk}-mean\left({X}_{j}\right))/sd\left({X}_{j}\right)$ forming a *d x n* matrix $\:Z\:=\:\left[{z}_{jk}\right]$. Finally, we define our *g* x *n* gene score matrix *G* $\:=\left[{g}_{ik}\right]$ as:

1 $$\:\begin{array}{c}{g}_{ik}\:=\:{\sum\:}_{j\:=\:1}^{d}{t}_{ij}{z}_{jk}\end{array}$$

P-values for each gene were generated by permuting the rows (drug labels) for $\:Z$ 1,000 times, and recomputing *G*, counting the number of times the permuted version of the score for each gene was less than the original.

#### Network propagation

We propagated our drug target scores to the larger set of genes comprising of the Reactome Functional (FI) gene interaction database^41^. We first limited the Reactome interactions to only those with a score > 0.95. Next, we converted it into an adjacency matrix $\:A=\left[{a}_{ij}\right]\:$where $\:{a}_{ij}=1$ if gene *i* is connected to gene *j* and 0 otherwise. We only considered genes that were part of the largest connected component. Note we are treating the graph as undirected whereas the Reactome FI framework also reports directionality for some interactions. We then normalized $\:A$ by the diagonal matrix $\:D$ containing the column degree:

2 $$\:\begin{array}{c}W\:=\:{D}^{-\frac{1}{2}}A{D}^{-\frac{1}{2}}\:\end{array}\:$$

For each patient we categorize the genes by the significance level of the gene scores. Top tier genes are P-value < 0.001, second tier < 0.01 and third is < 0.05. We define $\:{P}_{0}$ as a vector containing the most significant genes by gene score for a given patient, normalized to sum to 1.0. Fixing $\:\text{c}=0.7$, we solve:

3 $$\:\begin{array}{c}{P}_{k}\:=\left(1-\text{c}\right)\left(W{P}_{k-1}\right)+\left(c{P}_{0}\right)\:\end{array}$$

Until convergence^76^. In this approach, high scores of $\:{P}_{k}$ indicate a higher proximity to the selected genes and therefore more likely to be relevant to the drug response^40^. As the degree of genes in the interaction database can amplify the scores in $\:{P}_{k}$, we implemented an approach known as random degree preserving networks (RDPN) to generate a P-value adjusting for degree^77^. Specifically, the above procedure in (3) is computed for 1,000 re-wired networks where the degree is preserved. Empirical P-values are computed for each gene comparing how many times the original score (i.e. the corresponding element of $\:{P}_{k})$ is larger than those generated from re-wired graphs. Genes with a copy number alteration or mutation were prioritized for those genes with RDPN unadjusted P-values < 0.05. The subnetwork containing the top genes by score and alterations was formed using an adaptation of the STM Steiner tree approximation approach^78^. The resulting network plot was produced using Cytoscape^79,80^.

## Electronic supplementary material

Below is the link to the electronic supplementary material.

Supplementary Material 1

Supplementary Material 2

## Data Availability

The raw datasets generated and analyzed during the current study are available in the dbGaP repository (Accession number: phs003456.v1.p1), https://www.ncbi.nlm.nih.gov/projects/gap/cgi-bin/study.cgi?study_id=phs003456.v1.p1. Processed data as used in the analyses are publicly available at https://biodev.github.io/HNSCC/. Unedited microscopy images supporting Supplementary Figures S3 and S4 are available in FigShare: https://doi.org/10.6084/m9.figshare.28988237.v1.
