# Supplementary material for: Assessing individual head and neck squamous cell carcinoma patient response to therapy through integration of functional and genomic data
Source: Sci Rep. 2025 Jun 5;15:19742. doi: 10.1038/s41598-025-03111-7 (PMC12141454; doi:10.1038/s41598-025-03111-7)
Supplement: Supplementary file 1 — Supplementary Material 1 [file 41598_2025_3111_MOESM1_ESM.pdf]

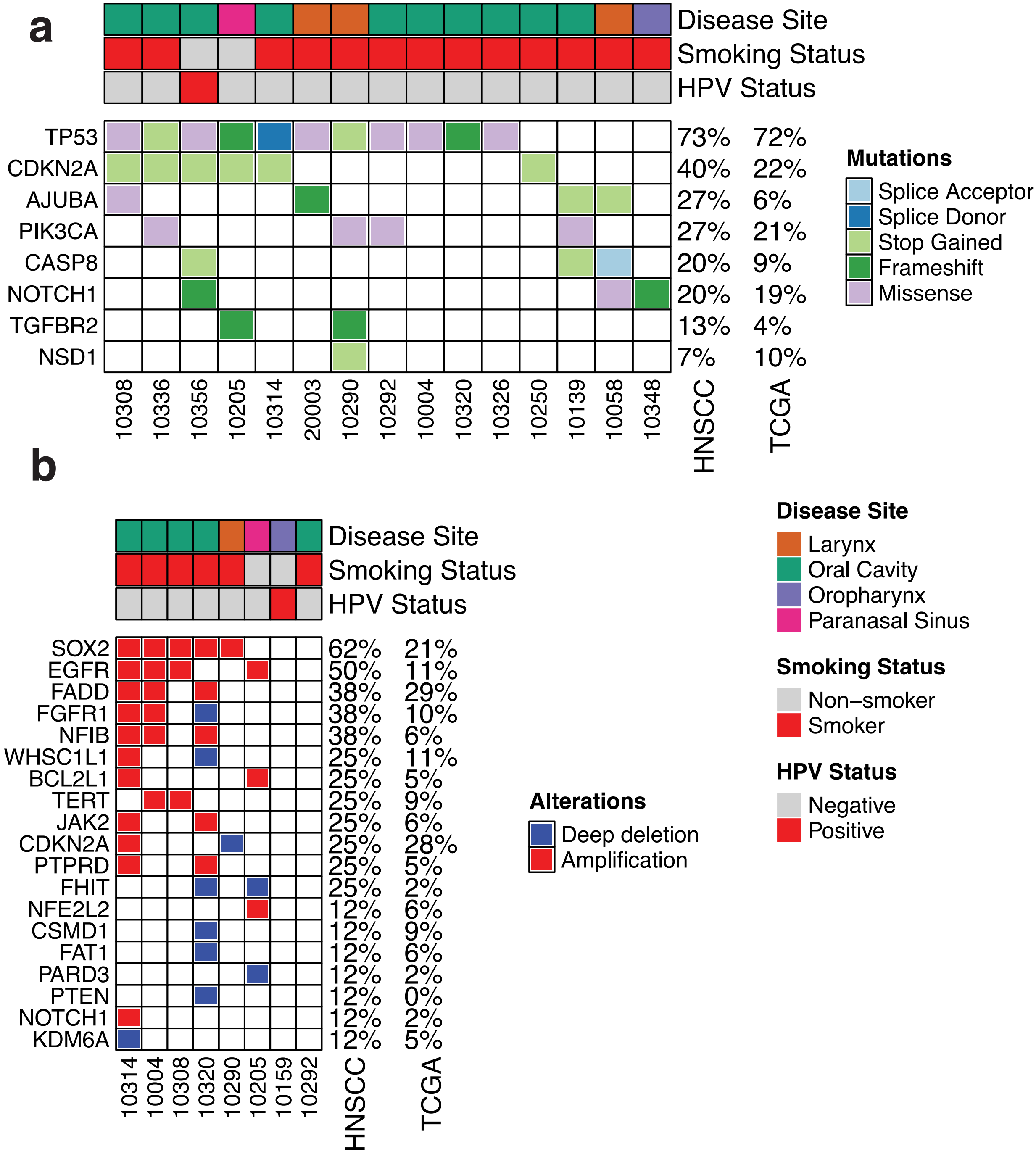
a
Disease Site
Smoking Status
HPV Status
TP53
CDKN2A
AJUBA
PIK3CA
CASP8
NOTCH1
TGFBR2
NSD1
73% 72%
40% 22%
27% 6%
27% 21%
20% 9%
20% 19%
13% 4%
7% 10%
10308 10336 10356 10205 10314 20003 10290 10292 10004 10320 10326 10250 10139 10058 10348
HNSCC
TCGA
Mutations
Splice Acceptor
Splice Donor
Stop Gained
Frameshift
Missense
b
Disease Site
Smoking Status
HPV Status
SOX2
EGFR
FADD
FGFR1
NFIB
WHSC1L1
BCL2L1
TERT
JAK2
CDKN2A
PTPRD
FHIT
NFE2L2
CSMD1
FAT1
PARD3
PTEN
NOTCH1
KDM6A
62% 21%
50% 11%
38% 29%
38% 10%
38% 6%
25% 11%
25% 5%
25% 9%
25% 6%
25% 28%
25% 5%
25% 2%
12% 6%
12% 9%
12% 6%
12% 2%
12% 0%
12% 2%
12% 5%
10314 10004 10308 10320 10290 10205 10159 10292
HNSCC
TCGA
Alterations
Deep deletion
Amplification
Disease Site
Larynx
Oral Cavity
Oropharynx
Paranasal Sinus
Smoking Status
Non–smoker
Smoker
HPV Status
Negative
Positive

Supplementary Figure S1. Summary of alterations relative to TCGA-HNSC. (**a**) Summary plot of mutations for the significantly mutated genes in TCGA (MutSigCV $q < 0.1$). Several relevant clinical variables are shown on the top. Gene mutations are indicated as colored rectangles ordered by frequency in our HNSCC cohort. Mutations are colored by type of mutation. Frequencies are shown on the right for both the HNSCC cohort as well as TCGA-HNSC. (**b**). Summary plot of copy number alterations for the putative driver genes in the TCGA driver peaks. The clinical variables shown on top are shared with (**a**). Genes are colored by type of alteration and ordered by frequency. Frequencies are shown on the right for both the HNSCC cohort as well as TCGA-HNSC.

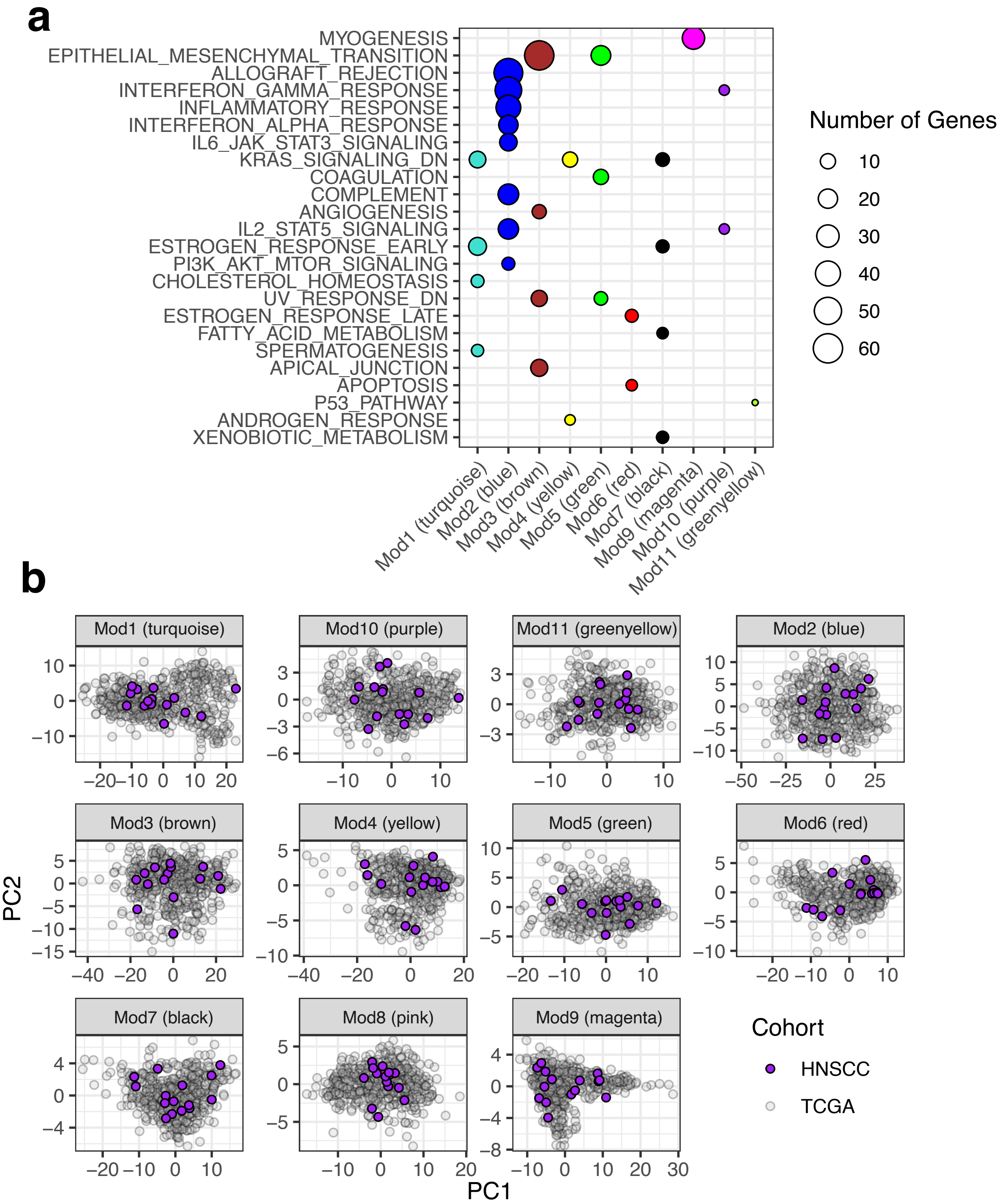
a
MYOGENESIS
EPITHELIAL_MESENCHYMAL_TRANSITION
ALLOGRAFT_REJECTION
INTERFERON_GAMMA_RESPONSE
INFLAMMATORY_RESPONSE
INTERFERON_ALPHA_RESPONSE
IL6_JAK_STAT3_SIGNALING
KRAS_SIGNALING_DN
COAGULATION
COMPLEMENT
ANGIOGENESIS
IL2_STAT5_SIGNALING
ESTROGEN_RESPONSE_EARLY
PI3K_AKT_MTOR_SIGNALING
CHOLESTEROL_HOMEOSTASIS
UV_RESPONSE_DN
ESTROGEN_RESPONSE_LATE
FATTY_ACID_METABOLISM
SPERMATOGENESIS
APICAL_JUNCTION
APOPTOSIS
P53_PATHWAY
ANDROGEN_RESPONSE
XENOBIOTIC_METABOLISM
Mod1 (turquoise)
Mod2 (blue)
Mod3 (brown)
Mod4 (yellow)
Mod5 (green)
Mod6 (red)
Mod7 (black)
Mod9 (magenta)
Mod10 (purple)
Mod11 (greenyellow)
Number of Genes
10
20
30
40
50
60
b
Mod1 (turquoise)
Mod10 (purple)
Mod11 (greenyellow)
Mod2 (blue)
Mod3 (brown)
Mod4 (yellow)
Mod5 (green)
Mod6 (red)
Mod7 (black)
Mod8 (pink)
Mod9 (magenta)
PC2
PC1
Cohort
HNSCC
TCGA

Supplementary Figure S2. TCGA-HNSC-derived WGCNA co-expression modules are both consistent with our HNSCC cohort and biologically relevant. (**a**) Significantly enriched (FDR < .05) Hallmark gene-sets (Y-axis) are shown with circles indicating the corresponding modules (X-axis). Dots are sized relative to the number of overlapping genes. Colors are derived from the module identifiers. (**b**) Shown are the first two principal component scores for each module shown separately with the module names indicated in the grey boxes on the top. Each point indicates a patient sample with the hollow grey points derived from TCGA and the purple points indicating HNSCC patient samples.

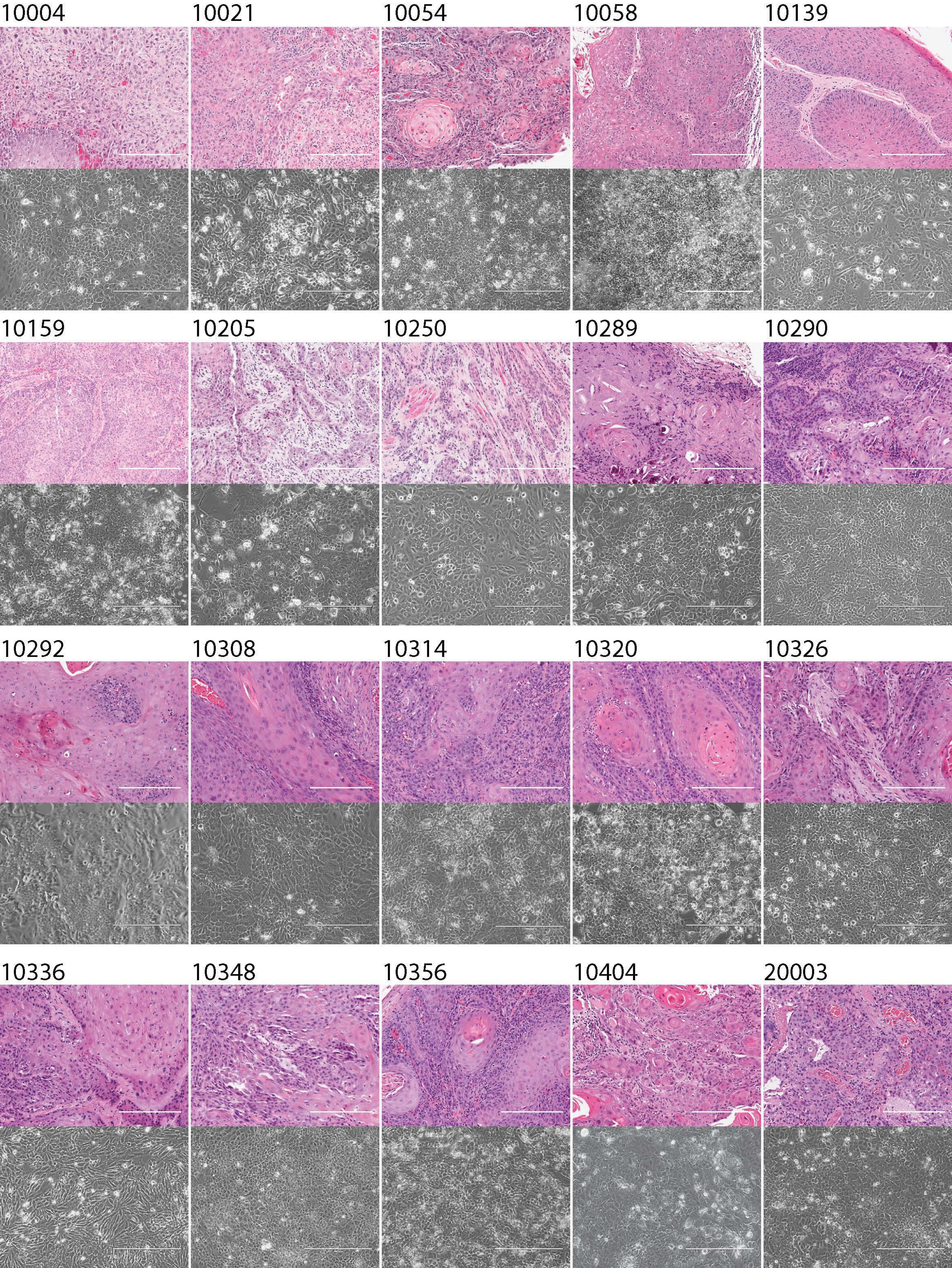
10004
10021
10054
10058
10139
10159
10205
10250
10289
10290
10292
10308
10314
10320
10326
10336
10348
10356
10404
20003

Supplementary Figure S3. Morphology of HNSCC tumors and derived primary cell lines. Photomicrographs of H&E stained FFPE tissue sections (top) and phase contrast imaging of primary culture derived from matched adjacent tissue (bottom). Scale bars equal 250um (H&E; 20x Objective) and 400um (cell culture; 10x Objective).

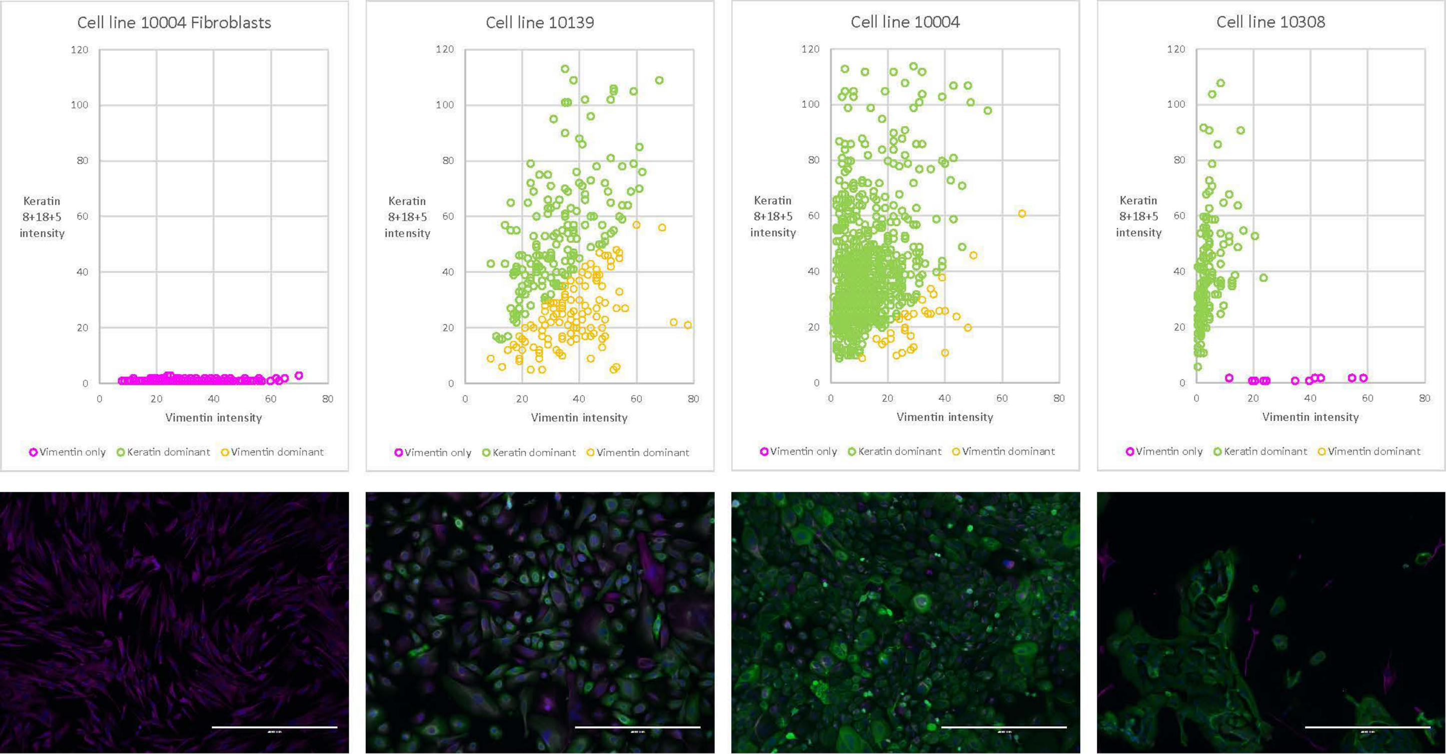
Cell line 10004 Fibroblasts
Cell line 10139
Cell line 10004
Cell line 10308
Keratin 8+18+5 intensity
Vimentin intensity
0
20
40
60
80
100
120
Vimentin only
Keratin dominant
Vimentin dominant

Supplementary Figure S4. Fluorescent Staining and Quantification of Characteristic Cell Types. A composite image of CY5 (vimentin, red), DAPI (nucleus, blue), GFP (K8/18, green), and TxRed (K5, pseudocolor green) channels are depicted alongside graphs of the signal intensities of each cell within the field. Cell lines were chosen to demonstrate the range of mesenchymal character expressed across the cohort. These include a purely fibroblastic line derived from 10004, an epithelial line with a high degree of mesenchymal character (10139), an epithelial line with lesser mesenchymal character (10004), and an epithelial line with minimal mesenchymal character and a subset of contaminating fibroblasts (10308). Epithelial (K5, K8/K18) and mesenchymal (vimentin) indirect immunostainings and analyses as described in Methods. 10x Objective. Scale bars equal 400um.

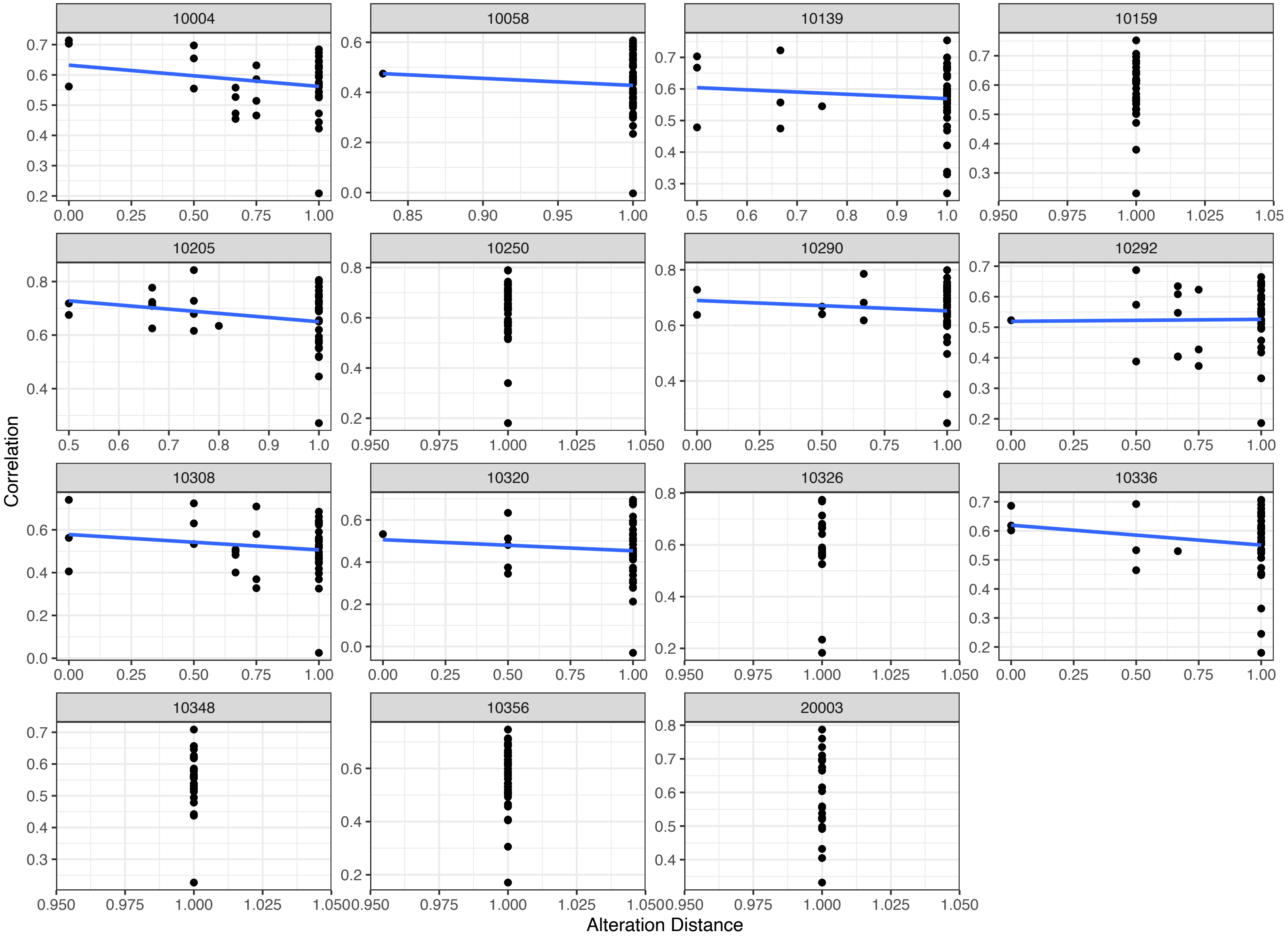

Supplementary Figure S5. Lower alteration distance between HNSCC patients and GDSC cells lines tends to result in higher correlation of drug response. For each patient sample, the Jaccard distance computed relative to the drug-associated genes from GDSC (X-axis) and the corresponding Pearsons correlation value (Y-axis) is shown for each cell line (points). The line of best fit is shown in blue.
